# Behavioral and electrophysiological evidence of reward processing deficits in repetitive negative thinking: Implications for depression

**DOI:** 10.1017/S0033291725102778

**Published:** 2026-01-14

**Authors:** Martino Schettino, Arianna Mastrocesare, Daniele Bomarsi, Ilenia Ceccarelli, Yuen Siang Ang, Diego A. Pizzagalli, Cristina Ottaviani, Sabrina Fagioli

**Affiliations:** 1 IRCCS Istituto Delle Scienze Neurologiche di Bologna, Italy; 2 Roma Tre University, Rome, Italy; 3 Sapienza University of Rome, Rome, Italy; 4 University of Bologna, Bologna, Italy; 5 Institute of High Performance Computing, Agency for Science, Technology and Research, Singapore; 6 Noel Drury, M.D. Institute for Translational Depression Discoveries, University of California, Irvine, CA, USA; 7 IRCCS Santa Lucia Foundation, Rome, Italy

**Keywords:** anhedonia, EEG, event-related potential, perseverative cognition, rumination

## Abstract

**Background:**

Anhedonia and rumination, a form of repetitive negative thinking (RNT), are key features of depression associated with poor treatment outcomes, chronic disease progression, and an increased risk of suicidality. Although their interaction is thought to sustain depressive states, the state-level mechanisms linking these symptoms remain poorly understood.

**Methods:**

In this multilevel, randomized within-subjects study, 62 individuals (n = 38 females) with varying levels of depressive symptoms completed the Probabilistic Reward Task (PRT) under two conditions: experimentally induced RNT and an active control. Concurrent electroencephalography was employed to assess electroencephalographic markers of reward functioning.

**Results:**

RNT significantly attenuated both reward response bias and feedback-related positivity (FRP) amplitudes, with the most pronounced effects in individuals with more severe depressive symptoms. These effects were not attributable to differences in task difficulty or perceptual cortical processing of PRT stimuli, supporting the specificity of RNT’s impact on reward-related processes.

**Conclusions:**

RNT may transiently disrupt behavioral and neural indicators of reward functioning. These findings suggest that cognitive states such as RNT can exacerbate or reveal the latent reward-processing deficits typically observed in individuals with anhedonia. This state-dependent sensitivity highlights the potential utility of targeting RNT to restore reward processing in depression.

## Introduction

Major depressive disorder (MDD) is the leading cause of disability among psychiatric disorders (Vos et al., 2020). MDD affects cognition, motivation, emotion regulation, and motor functions, along with neurovegetative symptoms (American Psychiatric Association, 2022). A core symptom is anhedonia – the reduced ability or lack of reactivity to pleasurable stimuli (Der-Avakian & Markou, 2012) – which is linked to greater depression severity (Spijker, Bijl, De Graaf, & Nolen, 2001), poorer responses to psychotherapeutic and pharmacological treatments (Uher et al., 2012), and delayed remission (McMakin et al., 2012).

Mounting evidence links depression and anhedonia to disruptions in reward functioning (e.g. Der-Avakian & Markou, 2012; Pizzagalli, 2014, 2022). Studies using the Probabilistic Reward Task (PRT), a signal-detection task assessing individuals’ ability to respond to and learn from rewards, show that individuals with depression exhibit reduced response bias toward the most frequently rewarded stimulus (Pizzagalli, Iosifescu, Hallett, Ratner, & Fava, 2008; Pizzagalli, Jahn, & O’Shea, 2005). This diminished reward response correlates with greater anhedonia severity (Bogdan & Pizzagalli, 2006) and is mirrored by a larger feedback-related negativity (FRN, Santesso et al., 2008). This event-related potential (ERP) component, typically observed ~200–300 ms post-feedback at fronto-central electrodes (Miltner, Braun, & Coles, 1997), has been increasingly reconceptualized as feedback-related positivity (FRP). This shift in reconceptualization reflects evidence that the apparent ‘negativity’ actually mirrors a diminished reward-related positivity in response to unfavorable outcomes (Kujawa, Smith, Luhmann, & Hajcak, 2013; Proudfit, 2015; Weinberg, Riesel, & Proudfit, 2014). Accordingly, in the present study, we refer to the variation in FRN amplitude as the FRP, consistent with prior depression research (e.g. Bogdan, Santesso, Fagerness, Perlis, & Pizzagalli, 2011; Whitton et al., 2016). The FRP originates from the dorsal anterior cingulate cortex and is associated with mesocorticolimbic BOLD activity (Carlson, Foti, Mujica-Parodi, Harmon-Jones, & Hajcak, 2011) and dopamine signaling (Holroyd & Coles, 2002; Santesso et al., 2009). In healthy individuals, the largest reward learning on the PRT corresponds to larger FRP (i.e. less negative FRN) to rich reward feedback (Santesso et al., 2008).

Notably, reward deficits in individuals with MDD are often viewed as trait-like characteristics (Kuhn et al., 2025; Pechtel, Dutra, Goetz, & Pizzagalli, 2013; Weinberg & Shankman, 2017). However, there is growing evidence that these deficits exhibit state sensitivity, fluctuating in response to contextual factors such as stress (Bogdan et al., 2011; Bogdan & Pizzagalli, 2006) and cognitive-affective processes such as rumination (Schettino et al., 2021).

Rumination is another central feature of depressive symptomatology contributing to the onset, maintenance, and poor prognosis of MDD and has been defined as a state of repetitive and passive focus on depressive symptoms and their possible causes and consequences (Nolen-Hoeksema, Wisco, & Lyubomirsky, 2008). Recently, rumination and related processes (e.g. worry, trauma-related memories) have been conceptualized as sharing core dimensions, including intrusiveness, repetitiveness, and uncontrollability. These features are collectively termed repetitive negative thinking (RNT, Ehring & Watkins, 2008). Evidence suggests that RNT’s transdiagnostic dimensions may be more predictive of depression and anxiety disorders than disorder-specific features (Spinhoven, Drost, van Hemert, & Penninx, 2015; Spinhoven, van Hemert, & Penninx, 2018).

RNT is thought to prolong the cognitive representation of environmental stressors and their associated physiological stress responses (Brosschot, Gerin, & Thayer, 2006; Ottaviani et al., 2016). Notably, blunted reward responses may stem from dysfunctional stress-reward systems’ interactions (Pizzagalli, 2014) via medial prefrontal cortex dopamine release and increased ventral tegmental area excitability (Cabib, Ventura, & Puglisi-Allegra, 2002; Cao et al., 2010; Lowes et al., 2021), ultimately leading to reduced functionality in mesolimbic striatal regions. Human studies have linked trait rumination to heightened mesocortical activity (Erdman et al., 2020; Lin et al., 2024), yet the state-dependent effects of RNT on reward processing are understudied.

It is plausible that RNT disrupts behavior by interfering with reward system functionality. However, it remains unclear whether core deficits in anhedonia (i.e. reward functioning) and rumination are mechanistically related in individuals with depressive symptomatology. Whitmer, Frank, and Gotlib (2012) found that rumination increased sensitivity to reward probabilities in both depressed and nondepressed individuals. Conversely, Hitchcock et al. (2022) reported reduced reinforcement learning performance during state rumination, independent of any concomitant reduction in attentional span. Other studies have found limited evidence of a direct pathophysiological relationship between state rumination and anhedonia-related reward deficits (Forthman et al., 2023; Park et al., 2022; Schettino et al., 2021).

Given the contrasting evidence, the present study employed a randomized controlled within-subjects multilevel design to examine the experimental effects of inducing state RNT on behavioral and electrophysiological reward-related markers in individuals with varying depressive symptomatology severity. Specifically, the effects of an experimentally induced RNT condition on reward response bias and the concomitant modulation of FRP amplitude were compared to those of an active control condition. We further investigated whether the RNT condition affected reward processing as a function of depressive symptom severity, focusing on the development of reward response bias and associated FRP amplitude to reward feedback. Finally, we assessed whether the RNT condition specifically modulated FRP amplitude to reward feedback without affecting other ERP components (i.e. N100 and P300).

## Materials and methods

### Participants

Participants are undergraduate students from Sapienza University of Rome. The sample included participants with varying severity of depression based on their scores on the Beck Depression Inventory-II (BDI-II, Beck, Steer, & Brown, 1996). Based on clinical cut-off scores for depression, participants scoring below 10 on the BDI-II were assigned to the Low DEP group (minimal depressive symptoms), while those scoring above 20 were assigned to the High DEP group (i.e. moderate-to-severe depressive symptoms) (Beck et al., 1996). Participants with BDI-II scores between 10 and 19 (mild depressive symptoms) were excluded. See Supplement S1 for exclusion criteria and power analyses. The study received ethical approval from the Institutional Review Board of the Department of Psychology (Ref. N. 740/2020), Sapienza University of Rome.

### Clinical scales

A set of questionnaires assessing sociodemographic (i.e. sex assigned at birth, age, ethnic identification, and level of education); lifestyle; and clinical information (i.e. alcohol and nicotine consumption, physical activity, and medications use) were administered. Besides the BDI-II, participants completed the Snaith–Hamilton Pleasure Scale (Snaith et al., 1995); the Ruminative Response Scale (RRS, Nolen-Hoeksema, 1991); the Perseverative Thinking Questionnaire (PTQ, Ehring et al., 2011); and the Penn State Worry Questionnaire (Meyer, Miller, Metzger, & Borkovec, 1990) (Supplementary S2).

### Experimental design and procedure

**Figure 1.**
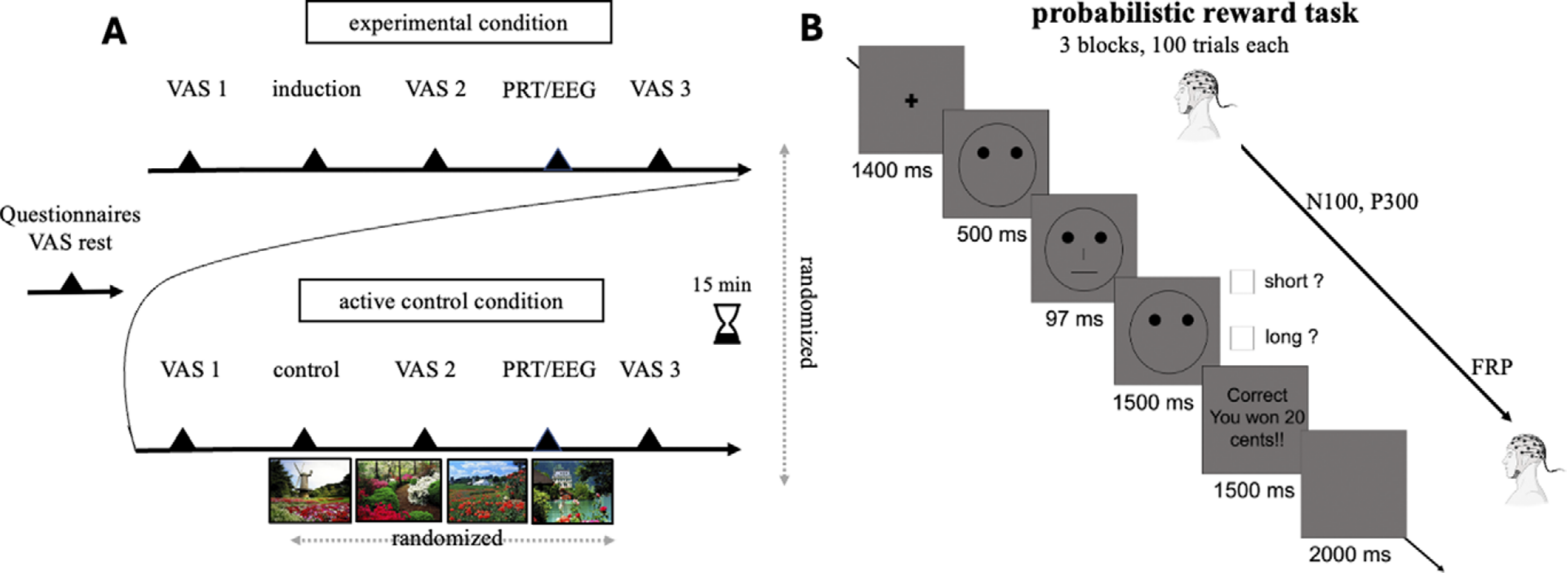
Experimental timeline of the study design (A). Participants underwent both the experimental and control conditions in a randomized order on the same day, with a 15-min break between sessions, during which they were only allowed to drink water. During the experimental condition, participants underwent the repetitive negative thinking induction, while during the control condition they underwent a corresponding active control induction. Visual analogue scales (VASs) were administered before, after, and at the end of each condition. EEG was registered from the scalp throughout the experiment. Feedback-related positivity (FRP) was detected after the reward feedback presentation to the rich stimulus, while N100 and P300 were detected after the presentation of the rich stimulus in the probabilistic reward task (PRT, B). The PRT required participants to respond to two different stimuli (long or short mouth or nose) presented on the screen, by pressing ‘m’ or ‘v’ buttons on the keyboard. Responses were followed by either a blank screen (non-reinforced trials) or by visual feedback indicating that they had won 20 cents (reinforced trials). One of the two stimuli was rewarded 3:1 time more frequently (i.e. the rich stimulus) than the other (i.e. the lean stimulus). The primary behavioral outcome variable in the PRT is the reward response bias, which is a signal detection-based measure of systematic preference to choose the most frequently rewarded stimulus.

The study used a randomized, controlled, within-subject design (Figure 1A). Participants were instructed to avoid consuming coffee or alcohol 1 hour prior to their experimental session. A 64-channel electroencephalography (EEG) cap was then applied to record electrophysiological activity from the scalp. Participants underwent, in random order, a 2-min experimental induction condition to elicit RNT and a 2-min active control condition where they described neutral pictures (see below). After each condition, participants performed the PRT, which lasted 21 min each. The entire experiment lasted 90 min, with a 15-min break in between sessions during which they were only allowed to drink water. Participants were then debriefed and compensated with the money they won during the PRT.

### RNT induction paradigm and control condition

Participants underwent a validated 2-min experimental induction condition aimed at eliciting RNT (e.g. Makovac et al., 2016) and a 2-min active control condition where they described a series of affectively neutral pictures. For the RNT condition, participants received the following instruction: ‘*Next, I would ask you to recall an episode that has happened in the past or something that may happen in the future; an event or an image that often intrudes in your mind repeatedly without you wanting it to. Please, take as much time as you need to recall such an episode in detail, for example considering its possible causes, consequences, and your feelings about it. Press the button whenever you are ready. Now, I ask you to tell me about that episode or image*’ (Supplementary Table S2 for qualitative descriptions of the thematic content generated during the induction).

For the active control condition, participants were instructed as follows: *‘Next, I would ask you to focus on the pictures that will be presented on the screen. Please take as much time as you need to focus on the pictures in detail, for example, on aspects such as shape, color, and the arrangement of flowers or trees within the picture. Press the button whenever you are ready. Now, I ask you to describe these pictures’* (Supplementary S3).

### Visual Analogue Scales

The content-independent features of RNT (adapted from the PTQ) were assessed in both conditions by the following four separated 100-point visual analogue scales (VASs): ‘Right now, how much are you: (1) distracted by your thoughts (i.e. past memories, future worries, personal problems)? (*distraction*); (2) having these thoughts go through your mind repeatedly? (*repetitiveness*)’; (3) having these thoughts come to mind without wanting them to? (*intrusiveness*); (4) stuck on these issues and unable to move on? (*being stuck*).’ These VASs were administered at rest, before and after the experimental/control condition and at the end of the PRT/EEG sessions (Figure 1A).

### Probabilistic Reward Task

The PRT assesses participants’ ability to adjust their behavior based on rewards, providing objective measures of reward learning (e.g. Pizzagalli et al., 2005).To minimize carry-over effects, two different versions of the PRT (mouth and nose) were administered before and after the experimental manipulation. Each run included three blocks of 100 trials. On each trial (Figure 1B), participants decided whether a long or short stimulus (mouth/nose) was presented on a cartoon face by pressing the ‘m’ or ‘v’ button on the keyboard. In reinforced trials, correct responses triggered visual feedback indicating a 20-cent reward; in nonreinforced trials, only a blank screen appeared. Unbeknownst to the participant, one stimulus (‘rich’) was rewarded three times more frequently than the other (‘lean’). The stimulus (mouth/nose) that was rewarded more frequently, the keys pressed for the short or the long mouth/nose, and the total rewards received were counterbalanced between and within participants, resulting in eight different combinations (Supplementary Table S1). The primary behavioral outcome variable was response bias, a signal detection-based measure of systematic preference for the most frequently rewarded stimulus (Supplementary S4). Control analyses included discriminability (Supplementary S5), accuracy, and reaction time (Supplementary S6).

### EEG acquisition

Continuous EEG was recorded (band-pass filter: 0.05–100 Hz; sampling rate: 500 Hz) using a BrainVision actiCHamp System (BrainProducts GmbH, Germany) with a set of 64 Ag/AgCl electrodes placed according to the 10/20 system and referenced to an electrode placed at the frontocentral (FCz) site with a cephalic (forehead) location as the ground. The vertical electrooculogram was recorded from the right eye using an infraorbital electrode. Horizontal eye movements were derived from electrodes near the outer canthi of the eyes. Electrode impedances were maintained below 5 kΩ.

### EEG analysis

Data were processed using Brain Vision Analyzer (Brain Products GmbH, Germany). A band-pass filter (0.1–40 Hz) was applied offline using an eighth-order Butterworth infinite-impulse response filter to decrease noise. All electrodes were referenced to the FCz electrode during data acquisition and rereferenced offline to the average of electrodes. Spherical spline topographic interpolation was used when necessary to handle noisy or missing channels, and a semiautomatic artifact-rejection method was used to remove muscle activity or other artifacts (Perrin, Pernier, Bertnard, Giard, & Echallier, 1987). An ocular independent component analysis was performed to eliminate artifacts related to eye movements, such as blinks and saccades. Components representing these ocular artifacts were identified and removed. EEG epochs were extracted beginning 200 ms before and ending 800 ms after the reward feedback presentation to the rich stimulus (i.e. the stimulus associated with the most frequent reward) on correct trials during Blocks 1 and 3 following previous works (e.g. Bogdan et al., 2011; Whitton et al., 2016). The FRP was scored manually for each subject at each site using a prestimulus baseline between −200 and 0 ms. The FRP was defined as the maximal peak occurring 200–400 ms after reward feedback presentation at fronto-central midline scalp sites, namely frontocentral (FCz), frontal (Fz), and central (Cz), where the FRP amplitude is typically largest (Gehring & Willoughby, 2002; Sambrook & Goslin, 2015). Based on previous findings (Pechtel et al., 2013; Santesso et al., 2008), we did not expect the induction to differentially affect FRP amplitudes to reward feedback across the three scalp sites. To determine whether the electrophysiological effects of RNT induction were specifically related to reward-related information processing, the N100 and P300 amplitudes time-locked to the rich stimuli were also analyzed (Supplementary S7). These ERPs inform on perceptual and attention allocation stages of information processing, respectively.

### Data analysis

Independent sample *t*-tests and chi-square analysis were performed to rule out preexisting group differences and potential confounders (i.e. body mass index, age, smoking). Preliminary checks confirmed that the assumptions of normality, linearity, and homogeneity of variances were met.

To evaluate the effectiveness of the RNT induction, mixed-model ANOVAs were conducted with *Condition* (RNT, Control) as a within-subjects factor, *Group* (High vs. Low DEP) as a between-subjects factor, and ΔVAS scores (postinduction minus preinduction) as the dependent variables.

PRT data were quality checked per established criteria (Pizzagalli, Jahn, & O’Shea 2005). The effect of the RNT induction (vs. control induction condition) on response bias was analyzed using mixed ANOVA with *Condition* (RNT, Control); *Block* (1–3) as within-subject factors; and *Group* (High, Low DEP) as between-subjects factor.

To examine the effects of the RNT induction on the FRP amplitude, a mixed ANOVA was conducted with *Condition* (RNT, Control) and *Site* (Fz, FCz, Cz) as within-subjects factors and *Group* (High vs. Low DEP) as between-subjects factor. Consistent with previous work, this primary analysis was done on the resulting FRP amplitude at Block 3 following PRT reinforcement schedule (Bogdan et al., 2011; Santesso et al., 2008). In addition, a secondary analysis tested FRP changes over time using separate mixed ANOVAs for each site with *Condition* (RNT, Control) and *Learning Phase* (early: Block 1, late: Block 3) as within-subjects factor and *Group* (High vs. Low DEP) as between-subjects factor. Simple effects analyses (Bonferroni-corrected) followed significant effects involving *Condition.* Statistical significance was determined based on 95% confidence intervals (CIs) of the marginal mean differences and corrected-*p* values.

Spearman’s correlations were used to test for associations between electrophysiological, behavioral, and subjective responses under the RNT induction.

## Results

Data from one participant did not pass the quality check for the PRT analyses and was excluded, resulting in a final sample of 61 participants. Additionally, seven participants had poor quality EEG data due to external noise and electrode artifacts, so the EEG analyses were conducted on a subsample of 54 subjects.

### Differences in clinical and sociodemographic variables between groups at baseline

Table 1 shows the mean and standard deviations for clinical and sociodemographic variables, as well as subjective levels of RNT at baseline. The High DEP group differed from the Low DEP group in sex distribution; thus, all subsequent analyses controlled for sex assigned at birth. The High DEP group reported significantly higher scores on the (1) brooding and depression subscales of the RRS, (2) PTQ, and (3) PSWQ, indicating a higher dispositional tendency to RNT. This pattern was also evident at the state level, as indicated by VAS scores at baseline, with participants in the High DEP group reporting higher levels of distraction, more intrusive, repetitive, and persistent thoughts.

**Table 1.** Sociodemographic, clinical, and baseline characteristic of the sample

Variable	High DEP (*n* = 24)	Low DEP (*n* = 37)	*t/χ* ^2^; *p*
**Demographics**			
Age, years	21.37 ± 3.16	20.92 ± 1.57	0.65; *p* = .52
%Women, *n*	12.2%, 20	11.0%, 18	7.45; *p* = .01
BMI, Kg/m^2^	23.11 ± 3.77	22.01 ± 3.21	1.70*; p* = .24
Education, years	13.78 ±1.34	13.46 ± 1.10	0.93*; p* = .35
Smoking status, y/n	12 yes, 14 no	12 yes, 15 no	0.16; *p* = .90
Cigarettes per day, *n*	2.01 ± 1.49	1.82 ± 1.11	0.35*; p* = .95
Alcohol, units/week	3.26 ± 2.13	3.60 ± 2.03	0.60*; p* = .54
Physical exercise, hours/week	1.61 ±0.49	1.54 ± 0.50	0.48; *p* = .62
**Clinical assessment**			
BDI	28.13 ± 7.64	5.32 ± 3.08	16.27*; p* < .0001
RRS reflection	10.43 ± 8.44	9.40 ± 3.36	1.28*; p* = .25
RRS brooding	13.39 ± 2.31	10.23± 2.47	4.86; *p* < .001
RRS depression	43.81 ± 16.17	28.15 ± 13.64	3.80; *p* < .001
PSWQ	57.22 ± 11.24	45.06 ± 10.58	5.28; *p* < .001
PTQ	41.57 ± 10.13	28.89 ± 8.07	3.82; *p* < .001
SHAPS	2.31 ± 3.11	3.78 ± 3.17	1.74; *p* = .08
**VAS (baseline)**			
Absent minded	41.74 ± 24.80	26.29 ± 26.24	2.24; *p* = .02
Repetitiveness	46.36 ± 29.03	24.29 ± 22.13	3.24; *p* = .002
Intrusiveness	50.83 ± 25.86	25.45 ± 19.54	4.23; *p* < .001
Stuck	40.42 ± 25.61	15.16 ± 14.57	4.61; *p* < .001

*Note:* Mean (SD) ± standard deviations are reported for all variables; BDI, Beck Depression Inventory; High DEP, individuals characterized by moderate/severe depression according to the Beck Depression Inventory-II; Low DEP, individuals characterized by minimal depression according to the Beck Depression Inventory-II; PSWQ, Penn State Worry Questionnaire; PTQ, Perseverative Thinking Questionnaire; RRS, Ruminative Response Scale; SHAPS, Snaith–Hamilton Pleasure Scale; VAS, Visual Analogue Scales.

### Effects of the induction on self-reported measures of RNT in individuals with High and Low DEP

**Figure 2.**
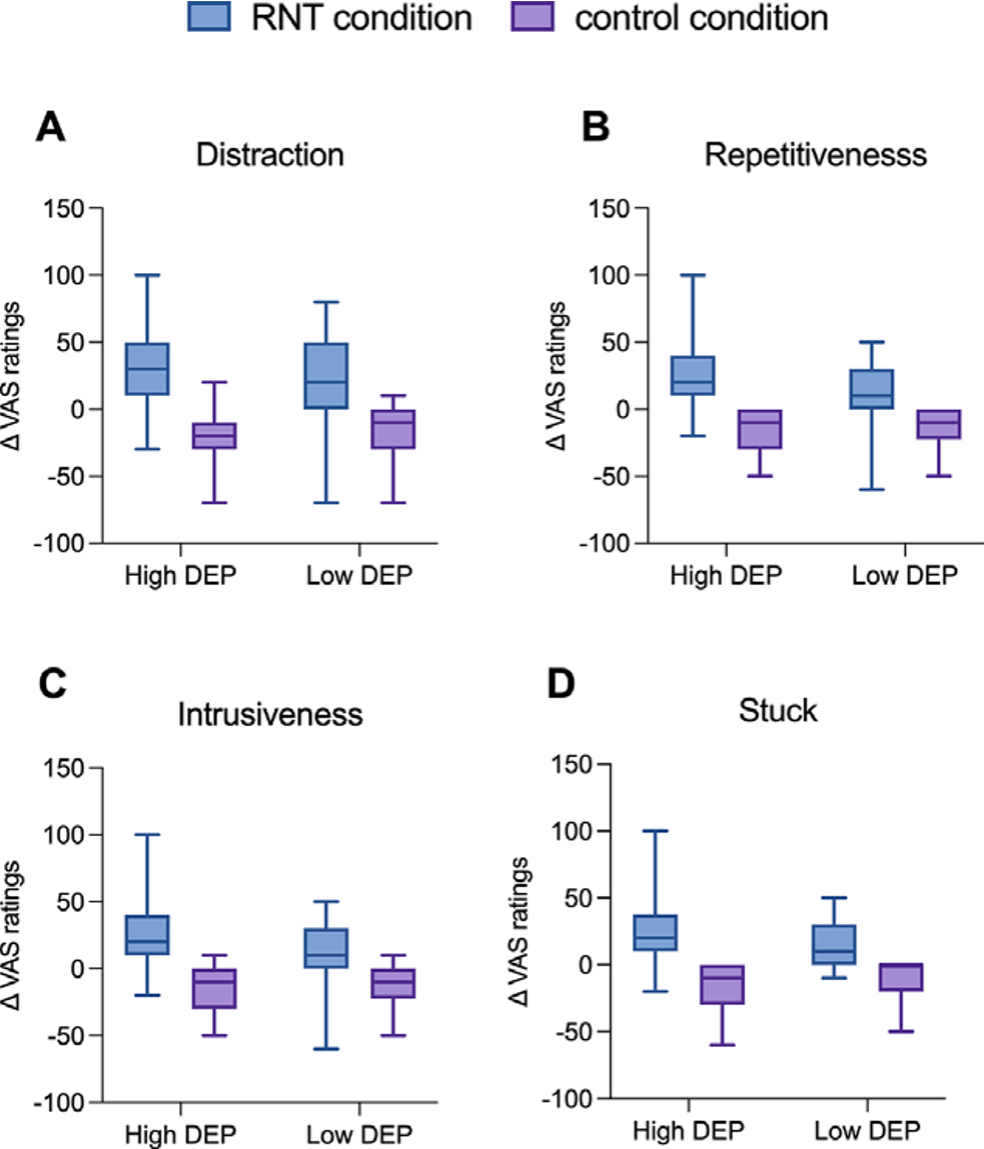
Visual analogue scales (VAS) rating changes for distraction (A), repetitiveness (B), intrusiveness (C), and stuck (D) during repetitive negative thinking and active control conditions in individuals with High versus Low DEP. *Note*: Error bars denote mean standard errors. High DEP, individuals characterized by moderate/severe depression according to Beck Depression Inventory-II scores, Low DEP, individuals characterized by minimal depression according to Beck Depression Inventory-II scores.

Main effects of *Condition* emerged for Δ distraction (*F*(1,55) = 62.20, *p <* .001, *η_p_* ^2^ = .51); Δ repetitiveness (*F*(1,53) = 102.69, *p <* .001, *η_p_* ^2^ = .65); Δ intrusiveness (*F*(1,53) = 56.64, *p <* .001, *η_p_* ^2^ = .51), and Δ stuck (*F*(1,49) = 56.62, *p <* .001, *η_p_* ^2^ = .53). Both High and Low DEP groups showed increased levels of VAS ratings in the RNT compared to the control condition (mean difference_distraction_ = 40.32, 95% CI = [30.08, 50.56], *p* < .001, Figure 2A; mean difference_repetitiveness_ = 40.54, 95% CI = [32.26, 48.56], *p* < .001, Figure 2B; mean difference_intrusiveness_ = 29.50; 95% CI = [21.64, 37.36], *p* < .001, Figure 2C; mean difference_stuck_ = 31.21, 95% CI = [22.87, 39.54], *p* < .001, Figure 2D). Ancillary analyses were additionally performed to assess whether group differences between participants with high and low levels of depression could be explained by the type of content generated in response to the RNT induction instructions (Supplementary S11 for full details; see also Supplementary Table S2).

### Effects of the induction on PRT performance in individuals with High and Low DEP

**Figure 3.**
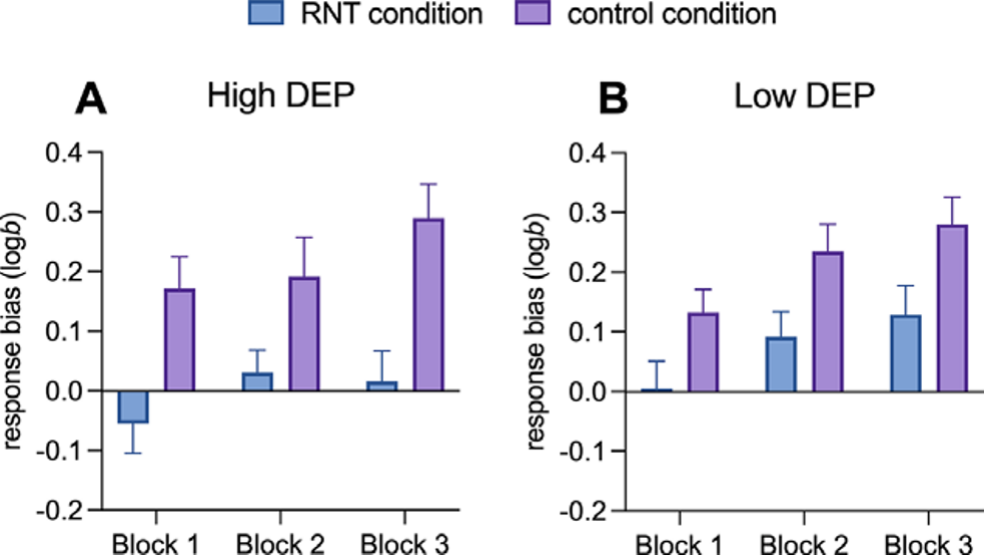
Development of response bias from Block 1 to Block 3 during repetitive negative thinking versus active control conditions in individuals with high (A) versus low DEP (B). *Note:* Error bars denote mean standard errors. RNT, repetitive negative thinking; High DEP, individuals characterized by moderate/severe depression according to Beck Depression Inventory-II scores, Low DEP, individuals characterized by minimal depression according to Beck Depression Inventory-II scores.

For the effects of the induction on PRT performance, a main effect of *Block* (*F*(1,116) = 12.08; *p <* .001, *ηp* ^2^ = .17) and a main effect of *Condition* (*F*(1,58) = 14.42; *p <* .001, *ηp* ^2^ = .20) emerged. Simple main effects analyses indicated that response bias increased from Block 1 to Block 3, with Block 1 showing a significantly lower response bias compared to Block 2 (mean difference = −0.08, 95% CI = [−0.15, −0.02], *p =* .027) and Block 3 (mean difference = −0.12, 95% CI = [−0.18, −0.06], *p <* .001). With respect to the main effect of *Condition*, simple main effects analyses indicated that the RNT induction reduced the response bias compared to the control condition (mean difference = −0.16, 95% CI = [−0.25, −0.08], *p* < .001, Figure 3A and B). No other significant effects emerged (all *ps* > 0.05).

No effects on discriminability emerged, indicating that response bias effects were not influenced by participants’ ability to discriminate between the big and little mouths or by task difficulty (Supplementary S8 and Figure S1). Higher accuracy and faster reaction times for the rich compared to the lean stimulus were observed only in the control condition (Supplementary S9 and Figure S1).

### Effects of the induction on FRP in individuals with High and Low DEP

For the effects of the induction on FRP amplitude to reward feedback during Block 3, a significant *Group* by *Condition* interaction was found for FRP (*F*(1,48) = 6.13; *p =* .016, *ηp* ^2^ = .11). Simple main effects analyses indicated that the interaction effect was due to smaller FRP amplitude (i.e. more negative) for the High DEP in the RNT condition relative to the control condition (mean difference = −1.33, 95% CI = [−2.52, −0.15], *p =* .027), but not for the Low DEP (mean difference = 0.49, 95% CI = [−1.37, 0.39], *p =* .269). No other significant effects emerged from this analysis.

**Figure 4.**
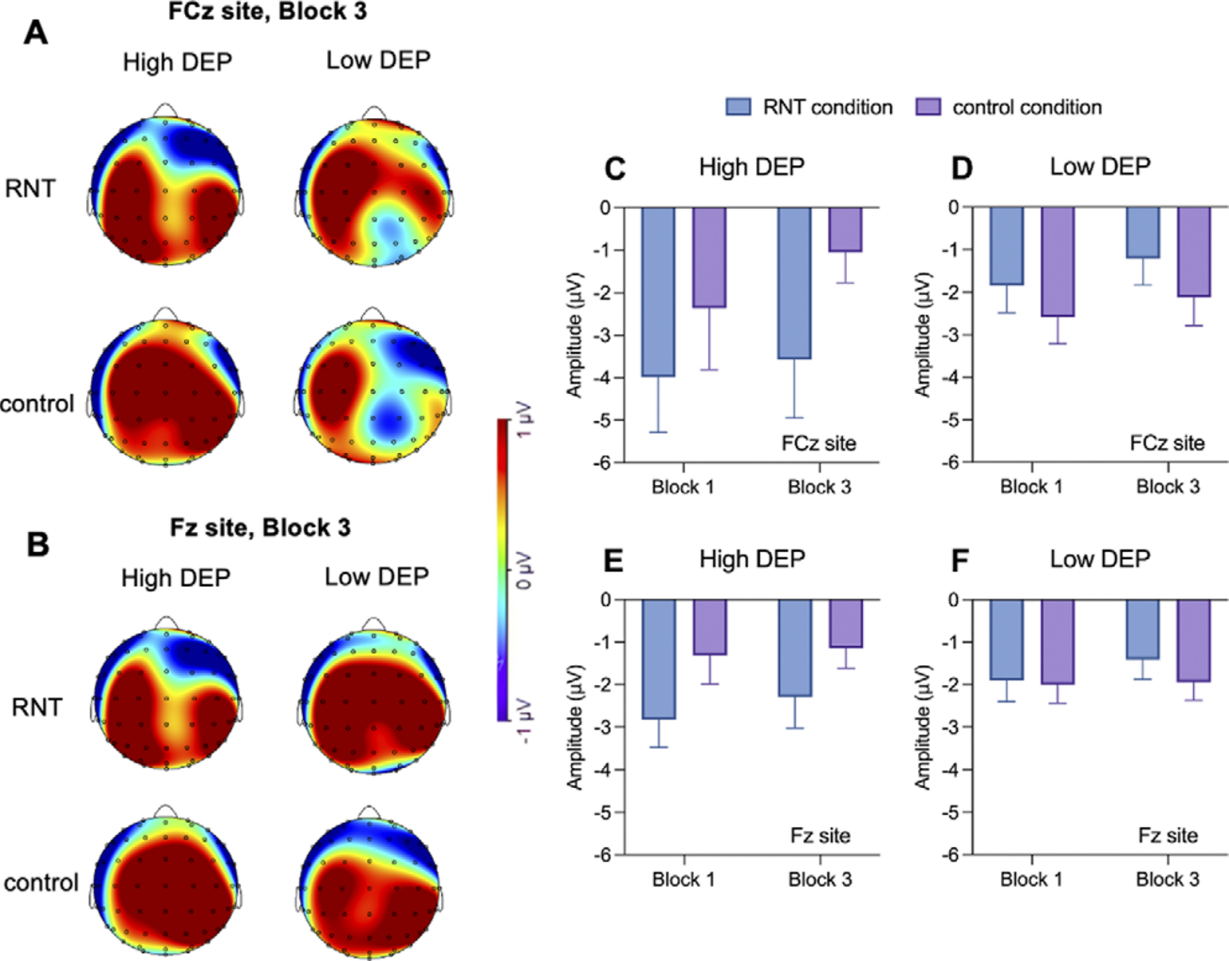
Topographic maps of the FRP FCz (A) and Fz (B) amplitude waving from 200 to 400 ms after the reward feedback presentation to the rich stimulus on correct trials during Block 3 for the repetitive negative thinking and active control conditions in individuals with High and Low DEP. Histogram of the averaged FRP amplitude in response to reward feedback during Block 3 for the repetitive negative thinking and control conditions in individuals with high (C and E) and low (D and F) DEP. *Note:* Error bars denote mean standard errors. RNT, repetitive negative thinking; High DEP, individuals characterized by moderate/severe depression according to Beck Depression Inventory-II scores, Low DEP, individuals characterized by minimal depression according to Beck Depression Inventory-II scores.

For the effects of the induction on the temporal evolution of the FRP to reward feedback, a significant *Group* by *Condition* interaction emerged for FCz (*F*(1,48) = 6.00; *p =* .018, *ηp* ^2^ = .10) and Fz (*F*(1,48) = 5 59; *p =* .022, *ηp* ^2^ = .10) and marginally for Cz (*F*(1,48) = 3.22; *p =* .079, *ηp* ^2^ = .07). Simple main effects analyses revealed that the interaction effect was due to smaller FRP amplitude at both sites for the High DEP in the RNT condition relative to the control condition (mean difference _FCz_ = −2.61, 95% CI = [−4.89, −0.32], *p* = .013, *p =* .025; mean difference _Fz_ = −1.52, 95% CI = [−2.75, −0.29], *p =* .016, Figure 4) but not for the Low DEP (mean difference _FCz_ = 0.87, 95% CI = [−0.82, 2.57], *p =* .306; mean difference _Fz_ = 0.29, 95% CI = [−0.62, 1.21], *p* = .522). In addition, a marginally significant *Condition* by *Learning Phase* interaction emerged for Fz (*F*(1,48) = 3.90; *p =* .054, *ηp* ^2^ = .07). FRP amplitude was smaller under RNT relative to the control condition in Block 1 (mean difference = −1.01, 95% CI = [−1.91, −0.11], *p =* .028) but not in Block 3 (mean difference = −0.21, 95% CI = [−1.04, −0.61], *p =* .604). No other significant effects emerged (all ps > 0.05). No main effects or significant interaction emerged for N100 and P300 (Supplementary S10 and Figure S2).

### Correlations between electrophysiological, behavioral, and subjective responses to the PRT after the RNT induction

**Figure 5.**
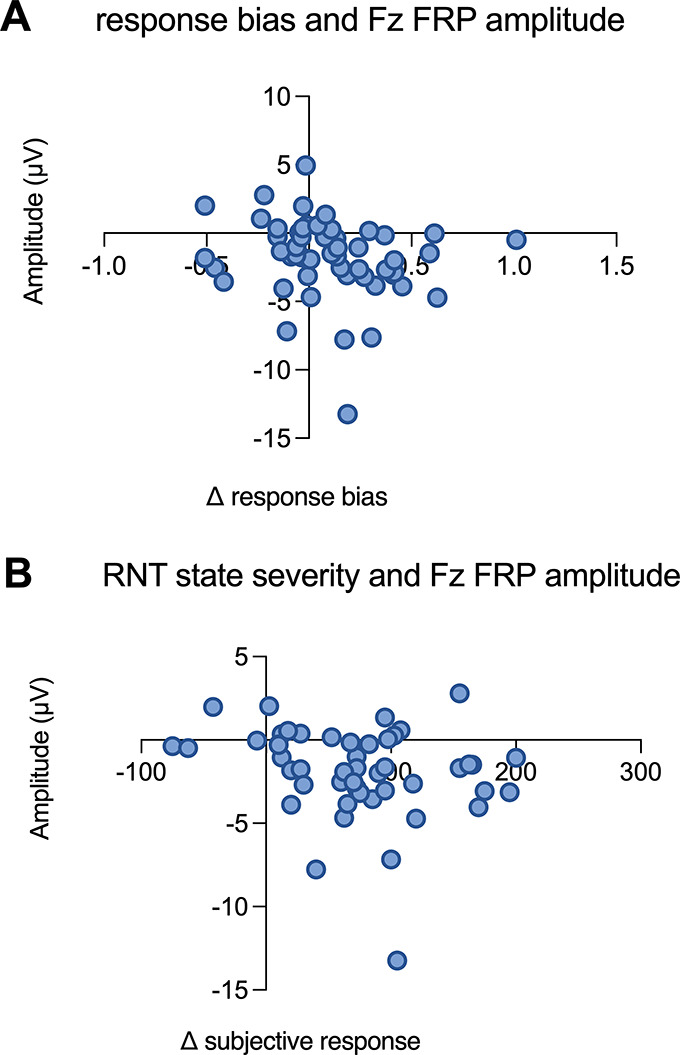
Scatterplots of the correlation between FRP Fz site amplitude and change scores in response bias (A) and severity of the subjective response to the RNT induction (B). *Note:* Change scores in response bias are calculated as response bias during Block 3 minus response bias during Block 1. The severity of subjective response to the RNT is calculated as the sum of ΔVASs (i.e. change scores on the VAS post-manipulation minus scores premanipulation for each VAS, i.e. distraction, repetitiveness, intrusiveness, and stuck).

Correlations were conducted between FRP amplitude at FCz and Fz in Block 3, changes in reward response bias, and the severity of the subjective response to RNT for a total number of five pairwise correlations. Reward response bias change was calculated as the difference between response bias in Blocks 3 and 1, where positive values indicate greater reward learning (e.g. Bogdan & Pizzagalli, 2006). The severity of the subjective response to RNT was computed as the sum of ΔVASs. Significant correlations emerged between FRP amplitude at the Fz site in Block 3 and (i) changes in reward response bias, *rho* = −0.31, *p* = .024, Figure 5A) and (ii) the severity of subjective response to RNT induction (*rho* = −0.28, *p* = .042, Figure 5B), indicating that a smaller FRP amplitude (i.e. more negative) correlates with a smaller reward response bias and a greater severity of subjective response to RNT. However, these analyses did not survive multiple comparison correction using Benjamini–Hochberg false discovery rate test.

## Discussion

Anhedonia and rumination are two clinically relevant hallmarks of the depressive phenotype. Although these symptoms may interact to sustain adverse depression-related outcomes (e.g. Rutherford, McDougle, & Joormann, 2023), relatively few studies have examined the mechanisms driving this interaction and its effects. This study concurrently examined behavioral and electrophysiological markers of reward learning in individuals with high and low depressive symptom severity during an experimentally induced state of RNT.

The RNT condition, compared to the active control condition, resulted in significantly lower reward-related responses at both behavioral and electrophysiological levels. Behaviorally, participants showed lower response bias toward the more frequently rewarded stimuli, suggesting that engendering RNT blunts reward learning, namely the ability to adjust behavior based on reward. This effect appeared to be particularly pronounced in individuals with high scores on the BDI-II and emerged in the context of no condition-related differences in discriminability (e.g. participants were not simply more distracted in the RNT relative to control condition). These findings are consistent with prior research showing decreased reward response bias in individuals with MDD, linked to their depressive symptoms’ severity, particularly anhedonia (i.e. Pizzagalli et al., 2005). Interestingly, in the current study, while all participants developed a response bias during the control condition, this pattern was disrupted under RNT, indicating that reward learning deficits, often seen as trait-like in depression, may also fluctuate in response to RNT patterns such as rumination.

Notably, and as mentioned above, analyses on discriminability scores revealed no significant differences between the RNT and control conditions or between groups, suggesting no global effects of RNT and depression severity on task difficulty. Analyses of accuracy and reaction time revealed increased scores for the rich stimulus type only during the control condition, suggesting that a specific impairment of reward-related behavior may occur during an RNT state.

Given that RNT is hypothesized to trigger and prolong physiological stress responses (e.g. Ottaviani, 2018), our findings align with evidence showing that acute stress blunts various aspects of reward processing (Schettino et al., 2024, for a recent meta-analysis). These include reduced reward responsiveness and learning (Bogdan & Pizzagalli, 2006) and other related subdomains, extensively investigated in relation to anhedonia, such as lower effort expenditure (Treadway, Bossaller, Shelton, & Zald, 2012) and diminished motivation for goal-directed behaviors (Grahek, Shenhav, Musslick, Krebs, & Koster, 2019). Preclinical studies similarly show that repeated stress induces anhedonic-like behaviors in animals (Schettino et al., 2024, for a meta-analysis). The RNT induction employed here mirrors stress-induction paradigms that evoke physiological activation through recall of personally relevant events (Ottaviani et al., 2016, for a meta-analysis). Although subjective response severity to the induction, in terms of these characteristics, was not linked to changes in response bias, it correlated with blunted ERP amplitude to reward feedback, suggesting that RNT’s pathophysiological impact on reward processing may be more evident at neural than behavioral levels.

Critically, electrophysiological analyses revealed smaller FRP to reward feedback presentation during RNT relative to the control condition, particularly in individuals with high BDI-II scores. These effects were not accompanied by changes in N100 and P300 amplitudes, suggesting a specific impact of RNT on cortical responses to reward feedback. Notably, smaller FRP amplitude to reward feedback has been found in individuals with current (Foti, Carlson, Sauder, & Proudfit, 2014) and remitted depression (Whitton et al., 2016) and correlates with suicidal ideation (Klumpp et al., 2023). We extended this literature by showing that an RNT state may acutely dampen FRP amplitude in individuals with high severity of depressive symptoms. This negative deflection, reflecting diminished reward-related positivity for reward outcomes that are worse than expected (Proudfit, 2015; Walsh & Anderson, 2012), may indicate a mismatch between reward expectations and outcome in individuals with depression. Supporting this, ecological data have linked momentary RNT with reduced sensitivity to reward despite intact motivation to pursue them (Schettino et al., 2021). Similarly, individuals with MDD show heightened anticipatory yet blunted consummatory reward network responses, potentially driven by rumination (Dichter, Kozink, McClernon, & Smoski, 2012). In this regard, smaller variations in FRP amplitude have been previously linked to reduced activation within the reward brain network, particularly in the ventral striatum, anterior cingulate cortex, and midfrontal cortex (Becker, Nitsch, Miltner, & Straube, 2014; Carlson et al., 2011). Additionally, FRP amplitude (and underlying midfrontal activation) appeared responsive to pharmacologic manipulation hypothesized to reduce phasic striatal dopaminergic responses (Santesso et al., 2009). Taken together, our findings indicate that state-level RNT can transiently worsen neural reward deficits typically considered trait-like in MDD, suggesting a dynamic interplay between cognitive states and enduring vulnerabilities. However, it remains unclear whether these observed deficits in reward learning are a direct consequence of the RNT interference within the neural reward systems, or if, alternatively, rumination primarily amplifies preexisting deficits characteristic of MDD.

The present findings warrant careful consideration for several reasons. First, contrary to expectations, no group differences in response bias emerged during the control condition, despite higher depressive symptoms in the High DEP group. One possibility is that the control induction unexpectedly enhanced positive affect or behavioral activation in this group. The control condition was designed to isolate the specific features of RNT by controlling for other potentially confounding factors, such as a general state of physiological activation or increased memory load. Notably, ancillary analyses indicated that individuals with depressive symptoms tended to engage in RNT at a more abstract level of construal compared to those without depressive symptoms. However, we lack information on potential group differences in the control condition, which may have been influenced by individual variability in visual imagery ability. Future studies should therefore incorporate qualitative assessments of participants’ subjective experiences during both RNT and control inductions to better characterize group-related differences in the cognitive and experiential processes underpinning RNT effects on reward processing. Second, there was a disproportionate number of male and female participants in the High and Low DEP groups, with fewer male participants in the High DEP group. While such gender differences could have influenced the results, this imbalance is commonly encountered in depression studies (e.g. Li, Zhang, Cai, et al., 2023). Importantly, sex assigned at birth did not emerge as a significant predictor in any of the analyses.

## Conclusions

Collectively, these findings suggest that transient cognitive states, such as RNT, may have pathophysiological and state-dependent effects, temporarily worsening or unmasking the reward-processing deficits commonly observed in individuals with MDD. This state sensitivity highlights the potential that targeting RNT could help restore reward processing. Supporting this idea, evidence suggests that behavioral activation or cognitive restructuring – core components of rumination-focused therapy (Watkins et al., 2011) – may not only improve mood but also enhance reward learning and neural responsiveness (Craske, Dunn, Meuret, Rizvi, & Taylor, 2024; Dichter et al., 2009; Kryza-Lacombe et al., 2021), ultimately help alleviate anhedonia (Webb, Murray, Tierney, Forbes, & Pizzagalli, 2023). These findings also support the development of dynamic or momentary interventions (e.g. mobile health tools or ecological momentary interventions) that monitor RNT (Funk, Kopf-Beck, Watkins, & Ehring, 2023) and deliver real-time strategies to disrupt it before it cascades into deeper reward dysregulation. Finally, as the FRP effect was more pronounced in individuals with higher BDI scores, this highlights the need for stratified interventions targeting those most vulnerable to reward-blunting effects from cognitive stressors.

## Supporting information

10.1017/S0033291725102778.sm001 Schettino et al. supplementary material Schettino et al. supplementary material

## Supplementary material

The supplementary material for this article can be found at http://doi.org/10.1017/S0033291725102778.
