# Supplementary material for: Behavioral and electrophysiological evidence of reward processing deficits in repetitive negative thinking: Implications for depression
Source: Psychol Med. 2026 Jan 14;56:e20. doi: 10.1017/S0033291725102778 (PMC12885348; doi:10.1017/S0033291725102778)
Supplement: Schettino et al. supplementary material [file S0033291725102778sup001.docx]

**Behavioral and electrophysiological evidence of reward processing deficits during repetitive negative thinking: implication for depression**

***Supplemental Information***

**Supplemental Methods**

**S1. Participants**

Exclusion criteria included: (a) history or presence of serious medical conditions (e.g., pacemaker, cardiac arrhythmia, hypertension, diabetes, endocrine, or metabolic disorders); (b) self-reported diagnosis of psychiatric disorders other than major depressive disorder; (c) neurological disorders, including traumatic brain injury or childhood neurological disorders; (d) use of drugs/medications (e.g., SSRI/SNRI, sedative-hypnotic or psychotropic medications, antiseizure drugs); (e) pregnancy or breast-feeding; and (f) BDI-II scores between 10 and 19 (mild depression). We based the calculation on preliminary results from a similar empirical study employing the PRT with a 2 x 2 x 3 mixed design (Schettino et al., 2021). To determine an adequate sample size, we conducted an a priori power analysis using G*Power (Faul et al., 2009). To achieve a power of 1-𝛽 > 0.90 and an effect size of 0.35 for a significant condition x group interaction, an adequate number of participants would be *n* = 60.

**S2. List of questionnaires**

The BDI-II (Beck et al., 1996) is a 21-item self-report questionnaire, to assess the severity of depressive symptoms. Items are rated on a 4-point scale from 0 (symptom absent) to 3 (severe symptoms). The minimum score is 0 and the maximum score is 63 with higher scores reflecting greater symptom severity: scores of 0-10 indicate minimal depression and characterize the low DEP group; scores of 11-19 indicate mild depression and represented an exclusion criterion; scores of 20-28 indicate moderate depression, and scores of 29-63 indicate severe depression and both characterize the High DEP group.

The Ruminative Response Scale (RRS, Nolen-Hoeksema, 1991) comprises 22 items that can be rated on a 4-point scale (from 1 = almost never to 4 = almost always) and reflects three aspects of rumination: brooding, reflective pondering and depression. Items can be distinguished into those focusing on the meaning of rumination, those capturing the feelings associated with a depressed mood, and those reflecting the tendency to ruminate on the causes and consequences of a depressed mood.

The Snaith-Hamilton Pleasure Scale (SHAPS, Snaith et al., 1995) is a 14-item scale providing an estimation of the degree to which a person can experience pleasure or anticipate a pleasurable experience in which ‘Disagree’ responses score 1 point and ‘Agree’ responses score 0 points. Thus, the score range is 0-14 where higher scores indicate a lower level of hedonic experience.

The Temporal Experience of Pleasure Scale (Gard et al., 2006) comprises 10 items measuring anticipatory pleasure and 8 items measuring consummatory pleasure, for a total of 18 items. The response format is a 6-point Likert scale (from 1 = very false for me to 6 = very true for me).

The Perseverative Thinking Questionnaire (PTQ, Ehring et al., 2011) consists of 15 items designed to capture the content-independent features of repetitive negative thinking. Items are rated on a scale ranging from ‘0’ (never) to ‘4’ (almost always).

The Penn State Worry Questionnaire (PSWQ, Meyer et al., 1990) is used to measure dispositional worry and includes 16 items reflecting excessive worry-related duration, uncontrollability, and distress. A 5-point answer scale (from 1 = not all typical of me to 5 = very typical of me) is used and the total score ranges from 16 to 80.

**S3. Active control condition description**

Participants were prompted to describe the pictures for a duration of two minutes. If they finished their description before the allotted time, the experimenter encouraged further elaboration by asking specific questions about the picture's details (e.g., “What colors do you notice in the picture?”).

**S4. PRT response bias**

Response bias is a signal detection-based measure that reflects a systematic preference for selecting the more frequently rewarded (rich) stimulus. A higher response bias results from a larger numerator (i.e., larger number of rich correct and lean incorrect) or from a smaller denominator (i.e., smaller number of rich correct and lean correct).

$$\log b= \frac{1}{2}\log\left( \frac{(Richcorrect + 0.5)* (Leanincorrect + 0.5)}{(Richincorrect + 0.5)* (Leancorrect + 0.5)} \right)$$

**S5. PRT discriminability**

Discriminability in the PRT provides information about the subjects’ ability to discriminate between the two target stimuli (big or little mouth or nose). Discriminability is influenced by physical differences between the stimuli and thus informs about task difficulty. According to the behavioral model of signal-detection theory (McCarthy and Davidson, 1979; Tripp and Alsop, 1999), discriminability is computed as:

$$\log d= \frac{1}{2}\log\left( \frac{(Richcorrect + 0.5)* (Leancorrect + 0.5)}{(Richincorrect + 0.5)* (Leanincorrect + 0.5)} \right)$$

A constant of 0.5 was added to each variable to allow for response bias and discriminability calculations in cases where any raw cell was equal to 0.

**S6. PRT accuracy and reaction time**

PRT accuracy indicates the absolute difference in the percentage of correct responses between rich and lean trials type, while reaction time (RT) measures the speed of responses in milliseconds.

**S7. Control analyses on N100 and P300**

To examine whether RNT affects the perceptual components of early information processing, N100 during the presentation of rich stimuli was analyzed (Vogel and Luck, 2000). Additionaly, to assess wheteher stimulus presentation affects subsequent attentional resource allocation, P300 was also examined (Polich, 2007). To do so, EEG epochs were extracted from 200 ms before to 800 ms after the presentation of rich stimuli. N100 and P300 components were manually scored for each subject at each site using a pre-stimulus baseline between -200 and 0 ms. Based on previous literature, N100 amplitude was defined as the most negative peak occurring 70–130 ms after stimulus onset, while P300 amplitude was defined as the most positive peak occurring 300–500 ms after stimulus onset (Gable and Harmon-Jones, 2013; Stim et al., 2023; Zhang et al., 2015).

**S8. Effects of RNT on PRT discriminability**

To test for the effects of RNT (vs. control condition) on the ability to distinguish between PRT target stimuli, discriminability was analyzed using a mixed ANOVA model with *Condition* (RNT, Control) and *Block* (1, 2, 3) as within-subject variables and *Group* (High, Low DEP) as a between-subject variable. No significant main effects or interactions involving Condition or Group factors emerged (all *ps* > 0.10) on discriminability, indicating that response bias effects were not influenced by individual differences in the ability to discriminate between the big and little mouths/noses or by task difficulty (Figure S1 A, B). A main effect of Block emerged from this analysis (*F*(1,116) = 4.12; *p* = .018, *ηp^2^* = .04) indicating higher discriminability in block 2 relative to block 1 (mean difference = 0.064, 95% *CI* = [0.014, 0.114], *p* = .029) and 3 (mean difference = 0.06, 95% *CI* = [-0.001, 0.123] *p* = .059). Discriminability did not differ significantly between blocks 1 and 3 (mean difference = -0.01, 95% *CI* = [-0.064, 0.059], *p*  = .999).

**S9. Effects of the induction of RNT on PRT accuracy and reaction time**

Accuracy and reaction time (RT) were analyzed separately using mixed ANOVA models, with *Condition* (RNT, Control), Block (1,2,3), and *Stimulus Type* (Rich, Lean) as within-subject factors. A significant Condition x Stimulus type emerged for both accuracy (*F* (1,59) = 14.55; *p* < .001, *ηp^2^* = .19) and RT (*F* (1,59) = 12.42; *p* < .001, *ηp^2^* = .17). Simple main effects analyses indicated that these interactions were primarily driven by higher accuracy (mean difference = 0.105, 95 % CI [0.075, 0.135], *p* < .001) and faster RT (mean difference = -50.20, 95 % CI [-67.022, -33.377], *p* <.001) for Rich stimuli relative to Lean stimuli in the control condition (Figure S1 C, D). In addition, a main effect of Stimulus type (*F*(1,59) = 43.07; *p* < .001, *ηp^2^* = .41) and a significant interaction Block x Stimulus type (*F*(1,59) = 12.73; *p* < .001, *ηp^2^* = .17) emerged for both accuracy and RT (*F*(1,59) = 30.90; *p* < .001, *ηp^2^* = .34; *F*(1,59) = 7.22; *p* < .001, *ηp^2^* = .10). Simple main effects analyses revealed overall faster RT for Rich relative to Lean stimuli, with RT decreasing progressively from block 1 to block 3 (Figure S1 E, F).

**S10. Effects of the induction of RNT on N100 and P300**

To examine the effects of the experimental induction of RNT on the amplitude of N100 and P300 during the presentation of rich stimuli in block 3, a mixed ANOVA model was conducted with *Condition* (RNT, Control) and *Site* (Fz, FCz, Cz) as within-subject factors and *Group* (High vs Low DEP) as between-subject factor. No significant main effect of Condition (*F*(1,49) = 0.43; *p* = .517, *ηp^2^* = .02) or Condition x Group (*F*(1,49) = 1.96; *p* = .168, *ηp^2^* = .04) or Condition x Site interactions emerged (*F*(1,96) = 0.607; *p* = .546, *ηp^2^* = .03) for N100 (Figure S2A, B). Similarly, no significant main effect of Condition (*F*(1,49) = 0.03; *p* = .851, *ηp^2^* = .00) or significant Condition x Group (*F*(1,49) = 0.62; *p* = .436, *ηp^2^* = .00) or Condition by Site (*F*(1,49) = 0.82; *p* = .442, *ηp^2^* = .00) interactions emerged for P300 (Figure S2 C, D).

**S11. RNT induction thematic content**

To explore the possibility that behavioral and electrophysiological effects of the RNT induction could, at least in part, depend on qualitative properties of the induced repetitive thinking, we categorized the content of participants’ RNT induction according to the taxonomy proposed by Watkins (2008). This taxonomy distinguishes between constructive and unconstructive repetitive thinking based on dimensions such as valence (positive vs. negative), temporal direction (future vs. past), and level of construal (concrete vs. abstract).

At the end of each RNT session, the experimenter briefly transcribed the content described by each participant. The content was then categorized according to (i) valence (positive, negative, or mixed) and (ii) temporal orientation (future, past, or mixed). Additionally, the experimenter rated the level of construal at the end of each session using a Likert scale in response to the question: “*To what extent did the participant describe the event in a highly unconstructive and abstract manner*?” The scale ranged from 0 (low/concrete) to 9 (high/abstract).

Following the suggestion of an anonymous reviewer and informed by Watkins’s taxonomy, we hypothesized that participants with high versus low depressive symptoms might differ in the valence, temporal orientation, and level of construal of their RNT content. To test these hypotheses, a chi-square analysis was conducted for temporal orientation and Wilcoxon signed-rank test was conducted for levels of construal. The valence variable was not analyzed due to insufficient variability, as only one participant recalled a positive event.

Results indicated no significant group differences in temporal orientation (future vs. past vs. mixed) of the RNT content ( χ^2^_(2)_ = 0.570, *p* = 0.752). However, levels of construal differed significantly between groups (*Z*_(45)_ = 2.110, *p* = 0.034) with participants in the high-depression group (*M* = 8.184, SD = 0.605) exhibiting more abstract RNT than those in the low-depression group (*M*= 7.321, SD = 1.558).

Although worry typically involves repetitive thinking about potential future threats and rumination about past events (Watkins et al., 2008), it is unsurprising that temporal orientation did not differ between groups. The transdiagnostic framework of RNT conceptualizes it as a process characterized by intrusiveness, repetitiveness, and uncontrollability, rather than by symptom-specific features such as temporal orientation (Ehring & Watkins, 2008). In contrast, previous evidence suggests that highly abstract (i.e., high construal) RNT is more strongly associated with psychopathological outcomes (e.g., Watkins & Moulds, 2005). Consistent with this evidence, participants with higher depressive symptoms in our study exhibited more abstract and unconstructive RNT.

These findings suggest that the observed behavioral and electrophysiological effects of RNT on reward processing may be, at least in part, modulated by qualitative aspects of RNT content, particularly its level of construal.

**Supplemental Tables**

**Table S1.** Counterbalancing Table

|  | **Assessment 1** | | | | **Assessment 2** | | | |
| --- | --- | --- | --- | --- | --- | --- | --- | --- |
| Subject | Rich | Key | Version | Winnings | Rich | Key | Version | Winnings |
| 1 | big | m | Mouth | 15.80 | little | v | Nose | 16.20 |
| 2 | little | m | Mouth | 16.20 | big | v | Nose | 15.80 |
| 3 | big | v | Mouth | 16.20 | little | m | Nose | 15.80 |
| 4 | little | v | Mouth | 15.80 | big | m | Nose | 16.20 |
| 5 | big | m | Nose | 15.80 | little | v | Mouth | 16.20 |
| 6 | little | m | Nose | 16.20 | big | v | Mouth | 15.80 |
| 7 | big | v | Nose | 16.20 | little | m | Mouth | 15.80 |
| 8 | little | v | Nose | 15.80 | big | m | Mouth | 16.20 |
| 9 | big | m | Mouth | 15.80 | little | v | Nose | 16.20 |
| 10 | little | m | Mouth | 16.20 | big | v | Nose | 15.80 |
| 11 | big | v | Mouth | 16.20 | little | m | Nose | 15.80 |
| 12 | little | v | Mouth | 15.80 | big | m | Nose | 16.20 |
| 13 | big | m | Nose | 15.80 | little | v | Mouth | 16.20 |
| 14 | little | m | Nose | 16.20 | big | v | Mouth | 15.80 |
| 15 | big | v | Nose | 16.20 | little | m | Mouth | 15.80 |
| 16 | little | v | Nose | 15.80 | big | m | Mouth | 16.20 |
| .. | .. | .. | .. | ... | .. | .. | .. | .. |

**Table S2.** Qualitative descriptions of thematic content categorized by temporal orientation (future vs. past vs mixed), emotional valence (negative vs. positive), and level of construal (0 = [low/concrete] to 9 = [high/abstract]) in participants with high (high DEP) and low (low DEP) levels of depressive symptoms.

|  | **High DEP** | | | **Low DEP** | | |
| --- | --- | --- | --- | --- | --- | --- |
| **Topic** | **Temporal orientation** | **Valence** | **Level of construal*** | **Temporal orientation** | **Valence** | **Level of construal*** |
| 1. Death of grandfather |  |  |  | Past | Negative | 8.5 |
| 1. Concern about traineeship |  |  |  | Future | Negative | 2.5 |
| 1. Fear of being alone and unloved |  |  |  | Future | Negative | 8 |
| 1. Intrusive thoughts about having cancer | Future | Negative | 9 |  |  |  |
| 1. Worry about providing for the family |  |  |  | Future |  |  |
| 1. Self-blame about what could have been done to prevent ex girlfriend leaving |  |  |  | Past | Negative | 7,5 |
| 1. Worry about an upcoming trip with boyfriend and sister |  |  |  | Future | Negative | 7,5 |
| 1. Intense argument with mother |  |  |  | Past | Negative | 8,5 |
| 1. Father’s job loss | Mixed | Negative | 8,5 |  |  |  |
| 1. Argument with boyfriend | Past | Negative | 8 |  |  |  |
| 1. Rejected a friend who confessed his feelings | Past | Negative | 8 |  |  |  |
| 1. Intrusive thoughts about mother’s cancer returning | Mixed | Negative | 9 |  |  |  |
| 1. Breakup with boyfriend | Past | Negative | 8 |  |  |  |
| 1. Breakup with boyfriend |  |  |  | Past | Negative | 8 |
| 1. Argument with boyfriend |  |  |  | Mixed | Negative | 8 |
| 1. Death of grandmother |  |  |  | Past | Negative | 8 |
| 1. Death of a friend | Mixed | Negative | 9 |  |  |  |
| 1. Return of ex-girlfriend after infidelity |  |  |  | Mixed | Negative | 8,5 |
| 1. Conflictual relationship with a friend | Mixed | Negative | 8 |  |  |  |
| 1. Self-harming thoughts following a breakup | Mixed | Negative | 9 |  |  |  |
| 1. Anxiety about the examination period | Future | Negative | 7,5 |  |  |  |
| 1. Mother’s surgery abroad | Mixed | Negative | 8 |  |  |  |
| 1. Mountain landscape causing melancholy |  |  |  | Past | Positive | 3,5 |
| 1. Episode in which a girl spoke about her depression |  |  |  | Past | Negative | 4 |
| 1. Anxiety about an upcoming exam |  |  |  | Mixed | Negative | 6,5 |
| 1. Fear of remaining with parents due to a difficult family situation |  |  |  | Mixed | Negative | 8,5 |
| 1. Anxiety about an upcoming exam |  |  |  | Mixed | Negative | / |
| 1. Therapy session in which the therapist asked a delicate question | Past | Negative | 8,5 |  |  |  |
| 1. Concern about university career | Mixed | Negative | 7 |  |  |  |
| 1. Rejection from a football team due to a physical characteristic | Past | Negative | 7 |  |  |  |
| 1. Risk of unplanned pregnancy | Future | Negative | 8,5 |  |  |  |
| 1. Fear of having missed a professional opportunity |  |  |  | Mixed | Negative | 6,5 |
| 1. Suicide of a family member | Past | Negative | 8,5 |  |  |  |
| 1. Fear of falling behind peers and not meeting personal standards |  |  |  | Mixed | Negative | 7 |
| 1. Concern about traineeship | Future | Negative | 8,5 |  |  |  |
| 1. Relationship status |  |  |  | Future | Negative | 6 |

*Notes.* Level of construal was evaluated on a scale from 0 = low/concrete to 9 = high/abstract. It was possible to derive qualitative descriptions of thematic content following the RNT induction for a subsample of 36 participants (n = 19 with high depressive symptoms and n = 17 with low depressive symptoms).

**Supplemental Figures**

**Figure S1**. Control analysis on discriminability scores from Block 1 to Block 3 during repetitive negative thinking condition and active control condition in individuals with High **(A)** vs Low DEP **(B)**. Control analyses on accuracy and reaction time for rich **(C, E)** and lean **(D, F)** stimuli.

**
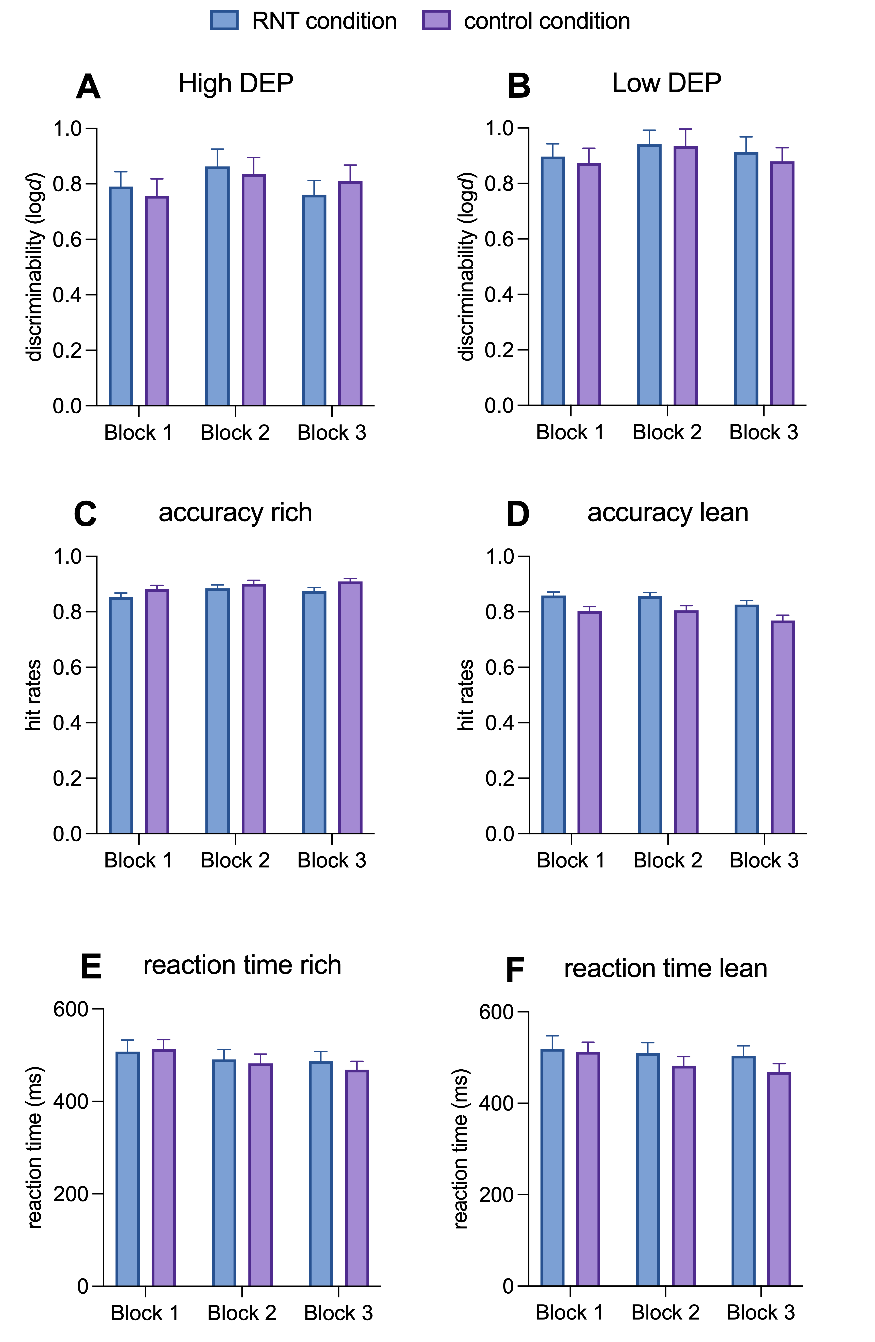
**

*Notes*: Error bars denote mean standard errors. RNT, repetitive negative thinking; High DEP, individuals characterized by moderate/severe depression according to Beck Depression Inventory-II scores, Low DEP, individuals characterized by minimal depression according to Beck Depression Inventory-II scores.

**Figure S2.** Control analysis of rich stimulus-locked N100 and P300 responses in Block 3 during the repetitive negative thinking and the active control conditions in individuals with High **(A, C)** vs Low DEP **(B, D)** at FCz, Fz and Cz sizes.


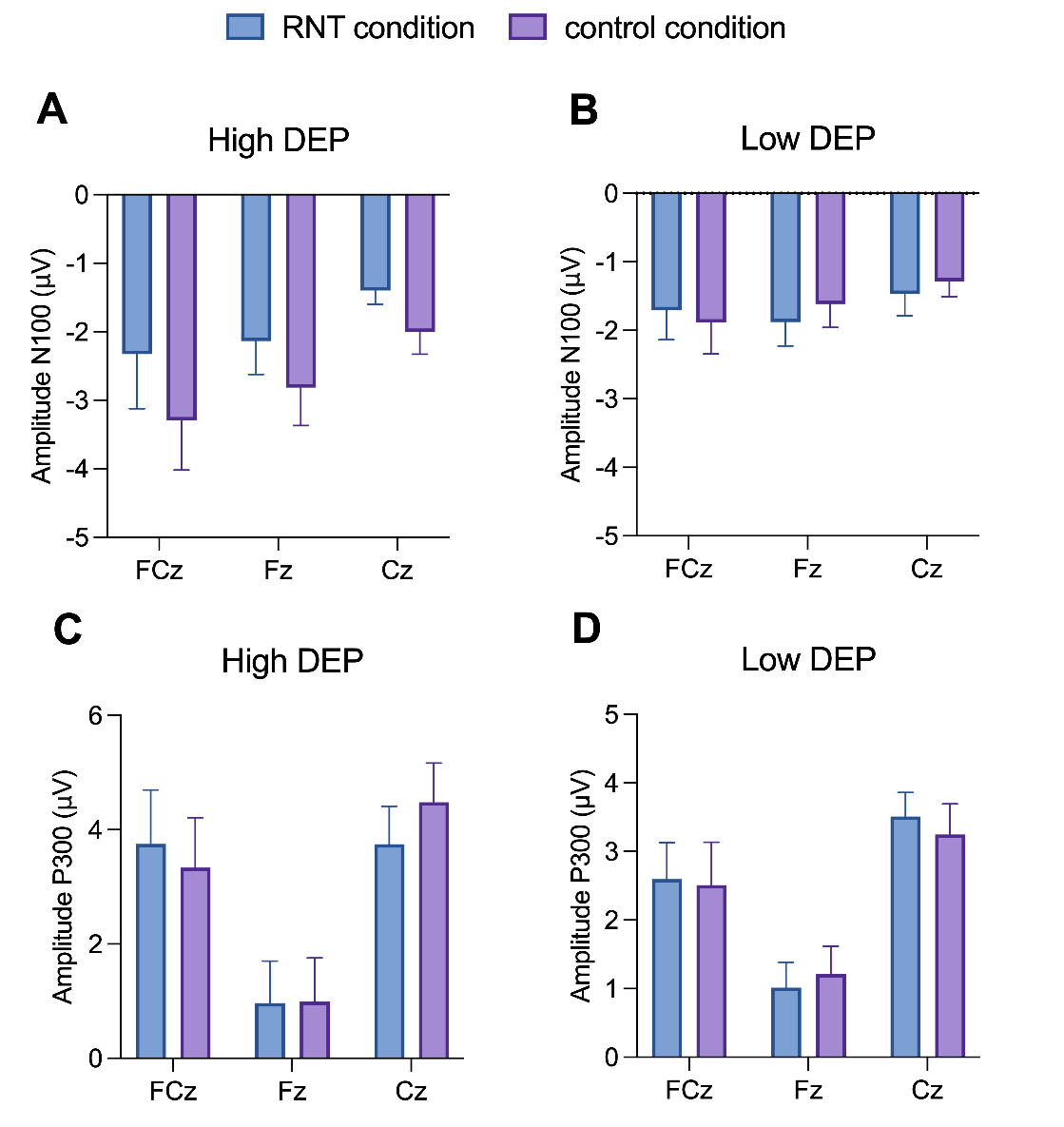


*Note*: Error bars denote mean standard errors. RNT, repetitive negative thinking; High DEP, individuals characterized by moderate/severe depression according to Beck Depression Inventory-II scores, Low DEP, individuals characterized by minimal depression according to Beck Depression Inventory-II scores.

**Bibliography**

Ehring, T., & Watkins, E. R. (2008). Repetitive Negative Thinking as a Transdiagnostic Process. *International Journal of Cognitive Therapy*, *1*(3), 192–205. <https://doi.org/10.1521/ijct.2008.1.3.192>

Faul, F., Erdfelder, E., Buchner, A., & Lang, A.-G. (2009). Statistical power analyses using G*Power 3.1: Tests for correlation and regression analyses. *Behavior Research Methods*, 41(4), 1149–1160. https://doi.org/10.3758/BRM.41.4.1149

Gable, P. A., & Harmon-Jones, E. (2013). Trait behavioral approach sensitivity (BAS) relates to early (<150 ms) electrocortical responses to appetitive stimuli. *Social cognitive and affective neuroscience*, *8*(7), 795–798. <https://doi.org/10.1093/scan/nss072>

Polich J. (2007). Updating P300: an integrative theory of P3a and P3b. *Clinical neurophysiology: official journal of the International Federation of Clinical Neurophysiology*, *118*(10), 2128–2148. <https://doi.org/10.1016/j.clinph.2007.04.019>

Stim, J. J., Maresh, E. L., Van Voorhis, A. C., Kang, S. S., Luciana, M., Collins, P., Sponheim, S. R., & Urošević, S. (2023). Neural abnormalities of reward processing in adolescents with bipolar disorders: An ERP study. *Biological psychology*, *183*, 108667. <https://doi.org/10.1016/j.biopsycho.2023.108667>

Vogel, E. K., & Luck, S. J. (2000). The visual N1 component as an index of a discrimination process. *Psychophysiology, 37*(2), 190–203. [https://doi.org/10.1017/S0048577200981265](https://psycnet.apa.org/doi/10.1017/S0048577200981265)

Watkins, E. R. (2008). Constructive and unconstructive repetitive thought. *Psychological Bulletin*, *134*(2), 163–206. <https://doi.org/10.1037/0033-2909.134.2.163>

Watkins, E., & Moulds, M. (2005). Distinct modes of ruminative self-focus: Impact of abstract versus concrete rumination on problem solving in depression. *Emotion*, *5*(3), 319–328. <https://doi.org/10.1037/1528-3542.5.3.319>

Zhang, X., Guo, Q., Zhang, Y., Lou, L., & Ding, D. (2015). Different timing features in brain processing of core and moral disgust pictures: an event-related potentials study. *PloS one*, *10*(5), e0128531. <https://doi.org/10.1371/journal.pone.0128531>
